# Eating and Obesity—The New World Disorder

**DOI:** 10.3390/nu5104206

**Published:** 2013-10-21

**Authors:** Neville Rigby

**Affiliations:** International Obesity Forum, 4 Moreton Place, London SW1V 2NP, UK; E-Mail: nevillerigby@aol.com

Obesity is not a new phenomenon. Paleolithic artefacts, some almost 35,000 years old, depict obesity in its classical gynoid form, suggesting that early hunter-gathers were not entirely safeguarded by the assumed Stone Age diet [1]. Nevertheless it has been convincingly argued by Boyd Eaton and others that the 21st century epidemic of non-communicable diseases (NCDs), including obesity, is attributable to mankind no longer enjoying the diet of our ancestors for which we remain genetically and metabolically programmed [2]. Even if our forebears seemed to revere obesity sufficiently to carve out stone “venuses”, it is still unclear if they were documenting a commonplace feature, although the frequency with which these venuses appear across thousands of years and even thousands of miles apart might suggest that obesity, in women at least, was not a complete rarity [3].

Our Stone Age forebears were in no position to take nutritional advice or make the right “lifestyle choices”, so given the apparent female propensity towards obesity, it would seem that nowadays the human race must be even more vulnerable given what has been widely regarded by many experts as today’s “toxic” food environment [4]; as Boyd Eaton has highlighted, this has only recently replaced natural food resources with a factory-supplied food chain combining a cornucopia of fats and sugar which were scarce in the past, but for which many of us appear to retain an intrinsic preference.

The modern-day pandemic of obesity, with its spectrum of NCD sequelae, is a truly new phenomenon, a transformation that can be charted across only a few decades; the World Health Organization estimates that since 1980 obesity has nearly doubled worldwide. The early harbingers of rising diabetes, heart disease, cancer and more recently even dementia are being vindicated with surprising rapidity [5]. The unprecedented scenario of up to one in three members of the human race in the grip of a seemingly relentless obesity epidemic has occurred during the marked social, economic and environmental changes that marked the second half of the 20th century [6]. Not least among the fundamental shifts is the altered nature of the human diet itself due to the radical transformation of the food chain. This is not the result of different personal preferences or individuals’ lack of understanding of “healthy choices”, but is marked by the rise and dominance of agribusiness conglomerates. Global corporations have spurred monoculture, modification of the genes of staple foods, globalized production and distribution systems which eliminating seasonal variation and cunningly contrived mass marketing strategies, which in turn have stimulated and sustained mass consumption. A new consumption paradigm featuring a predominance of foods that are bereft of natural nutritional value, but offer attractive, and some might say addictive, combinations of fats and sugars, has in its turn, fuelled growing overweight and obesity prevalence worldwide.

However obesity is also an indicator of population-wide weight gain. The new norm, with few exceptions, is a higher mean body mass index (BMI) for almost everyone, with the majority of adults in the Americas and European regions now being overweight, while WHO recommends lower cut off points to assess overweight for Asians. Even those within the normal weight spectrum are heavier than previous generations, given the rise in mean BMI. Hardly surprising then that the corollary is the emergence of the previously unknown phenomenon of significant levels of childhood overweight and obesity and the advent of what is now accepted as early onset type 2 diabetes.

Despite the oversimplistic cliché that excessive weight gain is simply the result of eating too much and exercising too little, there is much more to becoming obese than the old “greed and sloth” charge. Almost two decades of research since the discovery of leptin have done a great deal to confirm that the aetiology of obesity lies far deeper in the complex interaction of human genetics and metabolism and variable exogenous factors. No longer is it scientifically acceptable to simply point an accusing finger of blame at the obese—even if the obese themselves in interminable “reality” television programmes seem willing to castigate themselves and reinforce the prejudice of the minority of viewers who are a normal weight.

Outside factors range from the obvious influence of food availability and physical activity to less tangible elements such as in utero conditioning, epigenetics and hormone mimicking environmental pollutants. The repeated mantra of the global food and beverage industries seeks to shift responsibility onto the consumer to make healthy choices while enjoying their unhealthy products “as part of a balanced diet”. The strategy serves to distract from the commercial imperative to seek growth in sales and profits by constant promotion of their products.

The modern obesity epidemic did not begin with the headlines once shocking data became available. It gathered pace over the past 30–40 years. Obesity prevalence, once measured in single digit percentages, exceeds 20–30 per cent in many countries. Now we accept fatalistically that one third or more adults in the USA and Mexico is obese, at least one quarter of adults too in the United Kingdom, Canada, Chile, Hungary, Australia, and New Zealand [7]. Large parts of the troubled Middle East are becoming aware through the shock of type 2 diabetes that obesity, still regarded by some as a symbol of wealth, is not a sign of good health. In the Pacific Islands, the most obese populations in the world are still prevented from halting New Zealand’s undeniably cynical export of obesogenic fat residues called mutton flaps fearing further pressure from the World Trade Organization [8].

The World Health Organization has estimated that 1.5 billion adults are overweight and of those 0.5 billion are obese. Mapping the spread of obesity over time (first demonstrated by the Centers for Disease Control within the USA) demonstrates a pathway akin to a disease vector, become more obvious than ever elsewhere in the world with globalized markets for food and drink in effect exporting obesity—the “western disease” for which no-one has yet found a cure. Very few effective measures are available to counter obesity at a population level, while it has become obvious that societies need to confront the reality that obesity is placing severe demands on strained health systems. The remaining challenge of preventing childhood obesity is thwarted by an unchanged commercial environment that has fostered the present problem, with a new generation of parents who are themselves overweight or obese.

Although great concern in the present day food debate has been focused on the way genetically modified products have been introduced in many areas when the health consequences are unclear, the predominance of processed foods, confectionery and calorie-laden drinks over several generations has produced a widespread acceptance and even dependence on inert packaged foodstuffs with extended shelf lives, a surfeit of empty calories and a deficiency in fresh fruit and vegetable consumption. Moreover there is a lack of knowledge of the origins of food and the healthiest nutritional combinations.

The role of sugar specifically is again facing scrutiny both by the WHO’s expert nutrition advisory group and in the UK by the Scientific Advisory Committee on Nutrition, with implications not only for recommendations regarding weight gain, metabolic disorders and eating disorders, but as increasing evidence suggests, with implications for mental health including behavioural issues and a potential role in the growing problem of dementia [9].

It has been argued for some time that there may be an association between rising levels of obesity, disordered eating and eating disorders, and that there are some synergies to be explored in developing prevention strategies [10]. While it is clear that obesity is multi-factorial and that in many cases psychological factors do play a role in obesity, the extent of the obesity problem across a large section of the population confounds any attempt at generalisation other than a recognition that obesity may involve both physical and mental health concerns. The recent tardy acknowledgment by the American Medical Association that obesity is a disease was preceded in 1948 by the inclusion of obesity in the International Classification of Diseases. Despite this, some still seek to perpetuate the debate.

The motivation for disputing obesity’s evident damage to health is unclear. In the way the tobacco industry sought to deny the risks of smoking, the major corporations involved in the mass marketing of junk food and beverages have a vested interest in avoiding the “disease” label, for fear that litigation will follow. There has been a major shift towards focusing on NCDs, with growing involvement from the food sector including some controversial sponsorship arrangements [11].

Some see the UN initiative on NCDs as superseding WHO’s 2004 Global Strategy on Diet, Physical Activity and Health which faced intense lobbying and often undisguised opposition from large parts of the food and beverage sector. Since then even modest moves towards implementing the strategy taken by some authorities continue to face opposition. In the UK measures to curtail television advertising of food and beverages to children were resisted until regulation was enforced, attempts to introduce “traffic light” labelling were contested until major companies settled for a muted voluntary scheme, and major brands succeeded in seizing control of the national obesity strategy in a ironically named “Responsibility Deal”.

The United States Federal Trade Commission’s bid to introduce voluntary nutrition principles, tabled by its Interagency Working Group on Food Marketed to Children, were blocked by the food and beverage industries in 2011, leading the Institute of Medicine to highlight the importance of dealing with the problem of environmental cues instead of attaching blame and focusing on individual responsibility as an approach to dealing with obesity [12]. The regulatory action by Mayor Michael Bloomberg in New York City to moderate the excesses of sugar drink consumption by capping serving sizes to one pint rather than the quart servings promoted by soda companies prompted attacks from Coca-Cola and McDonald’s prior to legal action preventing the measure coming into force [13].

The thrust of many public health initiatives are in effect not only undermined but often sabotaged completely by commercial interests intent on thwarting effective controls on junk food and on marketing particularly to children. Whilst pledging to honour voluntary commitments, companies have switched from more expensive television advertising to exploit cheaper targeted behavioural marketing on the internet [14]. The WHO Director-General Dr. Margaret Chan has spoken out forcefully about the conflict of interest involving the food and beverage sectors influence: “Efforts to prevent noncommunicable diseases go against the business interests of powerful economic operators. In my view, this is one of the biggest challenges facing health promotion... it is not just Big Tobacco anymore. Public health must also contend with Big Food, Big Soda, and Big Alcohol. All of these industries fear regulation, and protect themselves by using the same tactics. Research has documented these tactics well: It includes front groups, lobbies, promises of self-regulation, lawsuits, and industry-funded research that confuses the evidence and keeps the public in doubt.” [15].

What is clear is that the failure to implement effective measures to improve dietary health makes it certain that the obesity epidemic will remain one of the biggest threats to health in the 21st century. Whether or not it is too late to overcome the challenge, only time will tell.
